# Bacteria associated with human saliva are major microbial components of Ecuadorian indigenous beers (*chicha*)

**DOI:** 10.7717/peerj.1962

**Published:** 2016-04-28

**Authors:** Ana L. Freire, Sonia Zapata, Juan Mosquera, Maria Lorena Mejia, Gabriel Trueba

**Affiliations:** Instituto de Microbiologia, Colegio de Ciencias Biologicas y Ambientales, Universidad San Francisco de Quito, Quito, Pichincha, Ecuador

**Keywords:** Lactic acid bacteria, Indigenous beer, Fermentation, Chicha, Microbiota, Artisanal fermented beverages, Streptococcus salivarius, Streptococcus mutans, Lactic acid bacteria, Fermented cassava, Ecuador, Chewed indigenous beer, Cassava, Saliva

## Abstract

Indigenous beers (*chicha*) are part of the indigenous culture in Ecuador. The fermentation process of these beers probably relies on microorganisms from fermented substrates, environment and human microbiota. We analyzed the microbiota of artisanal beers (including a type of beer produced after chewing boiled cassava) using bacterial culture and 16S ribosomal RNA (rRNA) gene-based tag-encoded FLX amplicon pyrosequencing (bTEFAP). Surprisingly, we found that *Streptococcus salivarius* and *Streptococcus mutans* (part of the human oral microbiota) were among the most abundant bacteria in chewed cassava and in non-chewed cassava beers. We also demonstrated that *S. salivarius* and *S. mutans* (isolated from these beers) could proliferate in cassava mush. *Lactobacillus* sp. was predominantly present in most types of Ecuadorian *chicha*.

## Introduction

The domestication of fermenting bacteria and yeast predated the domestication of animals and plants; ancestral hominids adapted to metabolize alcohol long before the Neolithic period (Carrigan et al., 2015). The organoleptic and psychotropic effects associated with the consumption of accidentally fermented fruits or cereals may have motivated early humans to replicate this process. Additionally, fermentation may have provided unintended benefits as fermenting bacteria may have reduced the risks of foodborne diseases in ancient societies (Nakamura et al., 2012; Lewus, Kaiser & Montville, 1991; Fooks & Gibson, 2002; Tesfaye, Mehari & Ashenafi, 2011); it is still unclear whether these microorganisms confer additional health benefits (McNulty et al., 2011). The use of alcoholic beverages has played a crucial role in the evolution of human societies (Joffe, 1998); nevertheless, very little is known about the process of domestication and evolution of these fermenting microorganisms (Libkind et al., 2011).

Many fermenting microorganisms have originated in the environment and food substrates (Martini, 1993) while others resemble microorganisms found in the human microbiome, suggesting human (skin or intestine) origins (Agapakis & Tolaas, 2012); in fact, some modern fermented dairy products contain intestinal bacteria (Walter, 2008).

Indigenous people from South America (such as in Ecuador) prepare a type of beer known as *chicha* which is made with either corn, boiled cassava or the fruit of the palm *Bactris gasipaes* (chonta); some cassava beers include an additional chewing step before the fermentation process. A recent report showed that bacteria present in chewed cassava beers were mainly *Lactobacillus* sp. (Colehour et al., 2014). We analyzed the microbial diversity (using culture dependent and culture independent techniques) in different types of Ecuadorian *chicha*.

## Materials and Methods

### Sample collection

Four samples of *chicha* (indigenous beer) from two geographical regions of Ecuador (Andean and Amazon regions) were collected. These samples included beer made with both chewed cassava (CC), mushed cassava (MC); mushed chonta (CB) and ground corn (CoB) (Table 1). The samples of CC and MC were purchased from the same household. All these products were obtained from rural communities. None of these beers were pasteurized, nor had they any commercial additives or preservatives. All samples were refrigerated (2–8 °C) after collection; a 2 mL aliquot of sample was stored at −20 °C, for molecular phylotyping.

**Table 1:** Description and site of collection of the different types of indigenous beers analyzed.

Main ingredient	Substrate scientific name	Geographical region	Site of collection	Time of fermentation
Chewed cassava	*Manihot esculenta*	Amazon	Puyo	3 days
Mushed cassava	*Manihot esculenta*	Amazon	Puyo	3 days
Chonta	*Bactris gasipaes*	Amazon	Tena	2 days
Corn (jora)	*Zea mays*	Highlands	Pifo	2 days

### Plate count of lactic acid bacteria (LAB)

A 20 mL aliquot of each sample was homogenized in 180 mL of a sodium citrate solution (10^−1^ dilution) and ten-fold dilutions were made in saline solution (NaCl 0.9%). One mL of each dilution was inoculated in MRS (pH 5) and M17 (pH 7, 0.5% dextrose) by pour plate method. Two incubation temperatures were used (37 and 43 °C) under aerobic and anaerobic conditions, for 3–5 days. The incubation time varied because of the different bacteria present on each product.

### Phenotypic characterization

Ten colonies (showing different morphology) were randomly picked from MRS plates from each sample. A subset of colonies showing characteristics of lactic acid bacteria (oxidase negative, catalase negative, Gram positive rods or cocci) was selected for molecular characterization (Table 2); 5 from CC, 6 from MC, 6 from CB and 8 from CoB. Strains were stored at −20 °C in MRS or M17 broth with 20% of glycerol.

**Table 2:** Bacteria isolated from the four beer samples. All the 25 strains were obtained by bacterial cultures in MRS and M17 and 16 S ribosomal gene from colonies was amplified and sequenced.

Sample	Isolate ID	Culture media	Growth condition	Identification (16S ribosomal RNA gene)
Chewed cassava beer	25 A2	MRS	Anaerobic	*Leuconostoc mesenteroides*
	25 C2	MRS	Aerobic	*Lactobacillus fermentum*
	25 E2	M17	Anaerobic	*Streptococcus mutans*
	25 F1	M17	Aerobic	*Lactococcus lactis*
	25H1	M17	Aerobic	*Streptococcus salivarius*
Mushed cassava beer	26 A1	MRS	Anaerobic	*Lactobacillus fermentum*
	26 B1	MRS	Anaerobic	*Lactobacillus fermentum*
	26 C2	MRS	Aerobic	*Lactobacillus fermentum*
	26 E2	M17	Anaerobic	*Streptococcus salivarius*
	26 F2	M17	Anaerobic	*Streptococcus salivarius*
	26 G1	M17	Aerobic	*Streptococcus salivarius*
Chonta beer	27 A1	MRS	Anaerobic	*Lactobacillus plantarum*
	27 B1	MRS	Anaerobic	*Weissella confusa*
	27 C1	MRS	Aerobic	*Weissella confusa*
	27 E1	M17	Aerobic	*Lactococcus lactis*
	27 F2	M17	Anaerobic	*Lactococcus lactis*
	27 G2	M17	Aerobic	*Lactococcus lactis*
Corn beer	61 B2	MRS	Anaerobic	*Lactobacillus casei*
	61 G1	M17	Anaerobic	*Leuconostoc mesenteroides*
	61 G2	M17	Anaerobic	*Lactobacillus plantarum*
	61 H1	MRS	Anaerobic	*Lactobacillus parabuchneri*
	61 I1	MRS	Anaerobic	*Lactobacillus paracasei*
	61 J1	MRS	Anaerobic	*Lactobacillus pantheris*
	61 K1	M17	Anaerobic	*Leuconostoc mesenteroides*
	61 L1	M17	Anaerobic	*Leuconostoc mesenteroides*

### Genotypic characterization of bacterial colonies

DNA was extracted from one colony using DNAzol Reagent (Life Technologies, Carlsbad, CA, USA) following manufacturer instructions and the DNA was stored at −20 °C until used. The 16S ribosomal RNA gene was amplified in 25 uL containing: 1X PCR buffer, 2.5 mM MgCl_2_, 0.25 mM dNTP’s, 0.2 uM 27F primer (5′-AGAGTTTGATCCTGGCTCAG-3′), 0.2 uM 1492R primer (5′-GGTTACCTTGTTACGACTT-3′) (Martin-Laurent et al., 2001), 0.5 U GoTaq Flexi DNA polymerase (Promega, Madison, WI, USA), 5 uL of sample DNA and Milli-Q water. The times and temperatures used for the amplification were: melting (94 °C, 1 min), annealing (56 °C, 30 s), elongation (72 °C, 30 s), this routine was repeated for 30 cycles, and final extension (72 °C, 10 min). Amplicons were subjected to gel electrophoresis (1% agarose gel), sequenced at Functional Biosciences (Madison, WI, USA) and DNA sequences analyzed using Seqmatch (Ribosomal Database Project: http://rdp.cme.msu.edu/) and submitted to GenBank; the accession numbers are KT722809–KT722833).

### High throughput sequencing analysis

In order to complement the culture-based protocols, we investigated the microbial diversity using FLX amplicon pyrosequencing. DNA was extracted from all beer samples using DNeasy Plant Mini kit (Qiagen) following manufacturer’s protocols, but instead of using AE buffer for elution, we used same volume of PCR Milli-Q water. DNA samples from four types of beer were sent to CD Genomics (Shirley, NY, USA), for 16S-based phylotyping. DNA was subjected to bacterial tag-encoded FLX amplicon pyrosequencing (bTEFAP) using primers 939F-5′TTGACGGGGGCCCGCAC3′ and 1492R-5′TACCTTGTTACGACTT3′. For fungal sequences we used ITSF-5′CTTGGTCATTTAGAGGAAGTAA3′. Resulting sequences (minimum length = 250 nucleotides) were trimmed and quality scored using USearch (http://drive5.com/); chimeras were detected using UCHIIME (http://drive5.com/) in de novo mode and were compared using BLASTn to a ribosomal database. Identity values were used to make assignments to the appropriate taxonomic levels: greater than 97% identity were resolved at the species level and between 95 and 97% at the genus level. The number of bacterial sequences we obtained were: 2,965 readings for CC, 3,320 for MC, 3,046 for B and 15,623 for CoB. For fungi we obtained 6,763 readings from CC, 6,925 from MC and 6,558 from CB. We did not carry out fungi analysis of CoB. All sequences were submitted to Sequence Read Archive and accession numbers are: SRP070493, SRS1299611, SRX1612367, SRR3202831, SRS1299612, SRX1612366, SRR3202830, SRS1299613, SRX1612365, SRR3202829, SRS1310202, SRX1600290, SRR3187397, SRS1310203, SRX1600289, SRR3187396, SRS1310204, SRX1600288, SRR3187395, SRS1310207, SRX1612364, SRR3202828, SRS1310208, SRX1600292, and SRR3202832.

### *Streptococcus salivarius* and *Streptococcus mutans* growth in cassava solution

To rule out the possibility of *S. salivarius* or *S. mutans* contamination, one colony of a pure culture of each bacteria (obtained from beers) was diluted in 25 mL of sodium citrate (2%) separately. Subsequently, 1 mL of this cell suspension was used to inoculate tubes containing 9 mL of sterile (autoclaved) chewed cassava solution (10%) and incubated at 37 °C under anaerobic conditions. A 100 μL aliquot from each incubated tube was extracted and plated in M17 (this was done by triplicate) at 0, 24, 48 and 72 h of inoculation. Results from each day were compared to determine the ability of these bacteria to grow in chewed cassava solution.

### Statistical analysis

We used Mann-Whitney U test to test whether *S. salivarius* and *S. mutans* were able to grow in cassava solution. Shannon indices were calculated using the formula *H* = −Σ*p_i_*log(*p_i_*), *p_i_* being the relative frequency of the abundance of each species found. Principal component analysis (PCA) of the bacterial species and abundance of the four beverages was performed using the software SPSS v21 (IBM Corp, Armonk, NY, USA).

## Results

### Characterization of bacterial isolates

Twenty-five bacterial isolates (cultured from the four beer types) were characterized by 16S rDNA sequencing showing 99–100% identity when compared with GenBank sequences (Table 2). The predominant bacterial species in all beers were *Lactobacillus fermentum* (16%), *Lactococcus lactis* (16%), *Leuconostoc mesenteroides* (16%), and *Streptococcus salivarius* (16%); followed by *Lactobacillus plantarum* (8%)*, Weissella confusa* (8%), *Lactobacillus casei* (4%)*, Lactobacillus pantheris* (4%)*, Lactobacillus parabuchneri* (4%)*, Lactobacillus paracasei* (4%) and *Streptococcus mutans* (4%). The most diverse bacterial composition (using culture-dependent techniques) found in CoB (6 bacterial species), followed by the CC (5 bacterial species), CB (3 bacterial species) and MC (2 bacterial species). Intriguingly, cassava beers contained human salivary bacteria: both CC and MC had *Streptococcus salivarius* while CC had also *S. mutans* (Table 2).

### High throughput sequencing analysis

The beer with greater diversity was CC (31 bacterial species), followed by CoB (26 bacterial species), CB (21 bacterial species), MC (20 bacterial species). The predominant bacterial species in CC were *Lactobacillus* spp. (40.9%) followed by human microbiota bacteria: *Streptococcus salivarius* (31.94%), *Streptococcus parasanguinis* (5.41%), *Streptococcus pneumoniae* (3.65%). The most prevalent bacteria in MC were *Streptococcus* spp. (83%) followed by *Lactococcus* sp. (9.32%); the majority of streptococci have been described as part of the human microbiota: *Streptococcus salivarius* (65%), *Streptococcus pasteurianus* (7.74%), and *Streptococcus parasanguinis* (3.47%). The most prevalent bacteria in CB were *Weissella confusa* (46%), *Weissella* sp. (20%), and *Lactococcus lactis* (9%). The dominant bacteria in CoB were *Weissella* sp. (19%) and *Lactobacillus plantarum* (12.5%), *Lactococcus garviae* (2.76%) *Lactobacillus brevis* (2.5 %) (Table 3). The dominant fungal species present in different beers analyzed was very similar; *Saccharomyces cerevisiae* was the most abundant comprising 92% of all the taxa detected (Table 4).

**Table 3:** Most predominant bacterial species (abundance of more than 0.1%) found by pyrosequencing analysis of samples from 4 types of *chicha*.

Bacterial species	CC	MC	CB	CoB	Cultured	Possible origins
*Bacillus amyloliquefaciens*	0.0	0.5	0.0	0.00	–	Environment
*Carnobacterium maltaromaticum*	0.0	0.0	1.0	0.1	–	Environment
*Enterobacter asburiae*	0.5	0.0	0.0	0.0	–	Environment
*Enterobacter cancerogenus*	0.5	0.0	0.0	0.0	–	Environment
*Enterobacter sp*	1.3	0.0	0.1	0.0	–	Environment
*Fructobacillus sp*	0.0	0.0	0.0	3.8	–	Vegetables
*Gluconacetobacter intermedius*	0.0	0.0	0.0	0.6	–	Fermented food
*Kluyvera ascorbate*	0.4	0.0	0.0	0.0	–	Human gut, food
*Lactobacillus brevis*	8.4	0.1	2.5	0.6	–	Environment, gut
*Lactobacillus camelliae*	0.0	0.0	0.0	7.3	–	Environment, gut
*Lactobacillus casei*	0.0	0.0	0.0	3.1	+	Environment, gut
*Lactobacillus delbrueckii*	8.0	0.0	0.0	0.0	–	Environment, gut
*Lactobacillus fermentum*	6.5	3.8	0.0	0.0	+	Environment, gut
*Lactobacillus harbinensis*	0.0	0.0	0.0	2.1	–	Vegetables
*Lactobacillus manihotivorans*	1.8	0.0	0.0	0.0	–	Vegetables
*Lactobacillus parabuchneri*	0.0	0.0	0.0	1.4	+	Oral microbiota
*Lactobacillus paracasei*	0.0	0.0	0.0	8.6	+	Environment, gut
*Lactobacillus paracollinoides*	0.0	0.0	0.0	16.0	–	Environment, gut
*Lactobacillus plantarum*	10.8	0.0	12.4	0.1	+	Environment, gut
*Lactobacillus sp*	3.4	0.0	0.7	1.3	–	Environment, gut
*Lactobacillus vaccinostercus*	1.2	0.0	0.2	0.0	–	Environment, gut
*Lactococcus garviae*	0.0	0.0	2.8	0.0	–	Fermented food
*Lactococcus lactis*	2.1	0.0	8.9	0.0	+	Environment, gut
*Lactococcus sp*	0.2	9.3	1.0	0.2	–	Gut
*Leuconostoc citreum*	0.0	1.5	1.2	0.0	–	Fermented food
*Leuconostoc lactis*	1.7	0.1	0.2	0.8	–	Environment
*Leuconostoc sp*	0.0	0.0	0.1	4.6	–	Vegetables
*Oenococcus kitaharae*	0.0	0.0	0.0	1.2	–	Vegetables
*Serratia sp*	1.0	0.0	0.0	0.0	–	Environment
*Streptococcus gallolyticus*	0.0	0.5	0.0	0.0	–	Oral microbiota
*Streptococcus oralis*	1.4	0.2	0.0	0.0	–	Oral microbiota
*Streptococcus parasanguinis*	5.4	3.5	0.0	0.0	–	Oral microbiota
*Streptococcus pasteurianus*	0.0	7.7	0.0	0.0	–	Human gut
*Streptococcus pneumoniae*	3.6	0.5	0.0	0.0	–	Human nasopharynx
*S. pseudopneumoniae*	0.5	0.0	0.0	0.0	–	Human nasopharynx
*Streptococcus salivarius*	32.0	65.0	0.0	0.0	+	Oral microbiota
*Streptococcus sp*	2.5	2.3	0.1	0.0	–	Human microbiota
*Streptococcus thermophilus*	1.2	2.59	0.0	0.0	–	Vegetables
*Streptococcus vestibularis*	0.4	0.8	0.0	0.0	–	Oral microbiota
*Weissella cibaria*	0.1	0.0	0.9	0.9	–	Vegetables
*Wbconeissella confusa*	0.5	0.1	45.9	25.3	+	Vegetables
*Weissella paramesenteroides*	0.5	0.0	0.1	0.0	–	Environment
*Weissella sp*	0.2	0.3	19.8	19.4	–	Vegetables

**Note:**

Chewed cassava, CC; mushed cassava, MC; chonta, CB; corn, CoB. Numbers indicate percentages and “+” indicates that bacterium recovered in culture.

**Table 4:** Most predominant fungal species found by pyrosequencing analysis of samples from 3 types of *chicha*.

Fungal species	CC	MC	CB	Possible origins
*Saccharomyces cerevisiae*	92.533	92.023	92.033	Vegetables
*Penicillium citrinum*	0.03	0.021	0.062	Soil
*Debaryomyces hansenii*	0.636	0.547	0.549	Sea water
*Hanseniaspora uvarum*	0.044	0.056	0.075	Vegetables
*Wallemia muriae*	0.118	0.115	0.137	Salty water
*Wallemia sp*	1.316	1.701	1.602	Salty water
*Aspergillus sp*	0.089	0.047	0.032	Soil
*Pichia kudriavzevii*	1.05	1.5	1.32	Vegetables
*Aspergillus versicolor*	0.104	0.138	0.135	Soil
*Pichia burtonii*	0.118	0.123	0.107	Vegetables
*Hyphopichia burtonii*	0.089	0.067	0.073	Starch substrates
*Cyberlindnera sp*	0.532	0.54	0.545	Waste deposits
*Pichia sp*	0.044	0.04	0.054	Soil
*Saccharomyces bayanus*	0.104	0.132	0.096	Vegetables
*Galactomyces sp*	3.149	2.908	3.133	Rumen, fermented food
*Pichia fermentans*	0.044	0.042	0.047	Vegetables

**Note:**

Chewed cassava, CC; mushed cassava, MC; chonta, CB. The numbers indicate percentages.

### Growth of *S. salivarius* and *S. mutans* in cassava solution

**Figure 1:**
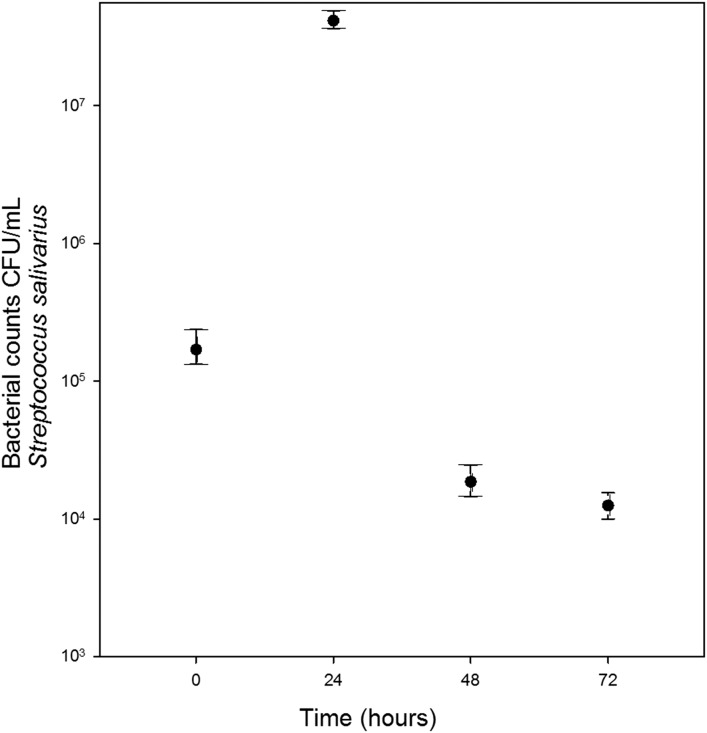
Growth of *S. salivarius* in sterile chewed cassava solution. There is a significant increase in CFU (Mann-Whitney U test) at the 24 h of incubation compared with those at inoculation time (0 h).

**Figure 2:**
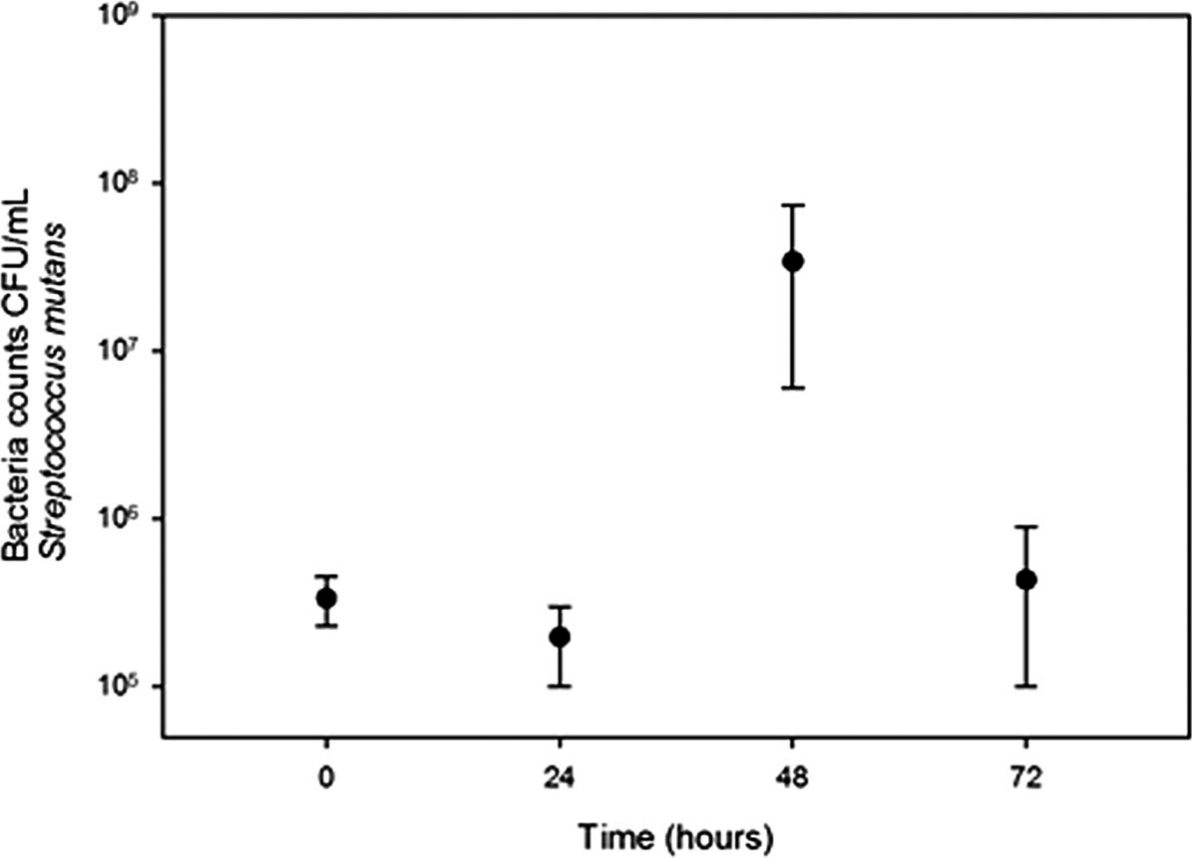
Growth of *S. mutans* in chewed cassava solution. There is a significate increase in CFU (Mann-Whitney U test) at the 48 h of incubation compared with those at the inoculation time (0 h).

*Streptococcus salivarius* (Fig. 1) and *S. mutans* (Fig. 2) grew in chewed cassava solution. After 48 h of culture (*S. salivarius*) and 72 h (*S. mutans*), the bacterial counts went down.

### Diversity estimations

CC was the beverage with the most species diversity (H = 1.06, E = 0.71), followed by CoB (H = 0.94, E = 0.66), CB (H = 0.71, E = 0.54), and MC (H = 0.59, E = 0.45). The evenness values followed the same pattern and suggest that CC is also the most heterogeneous in terms of species (Hayek & Buzas, 2010; Pielou, 1966).

### Principal component analysis

**Figure 3:**
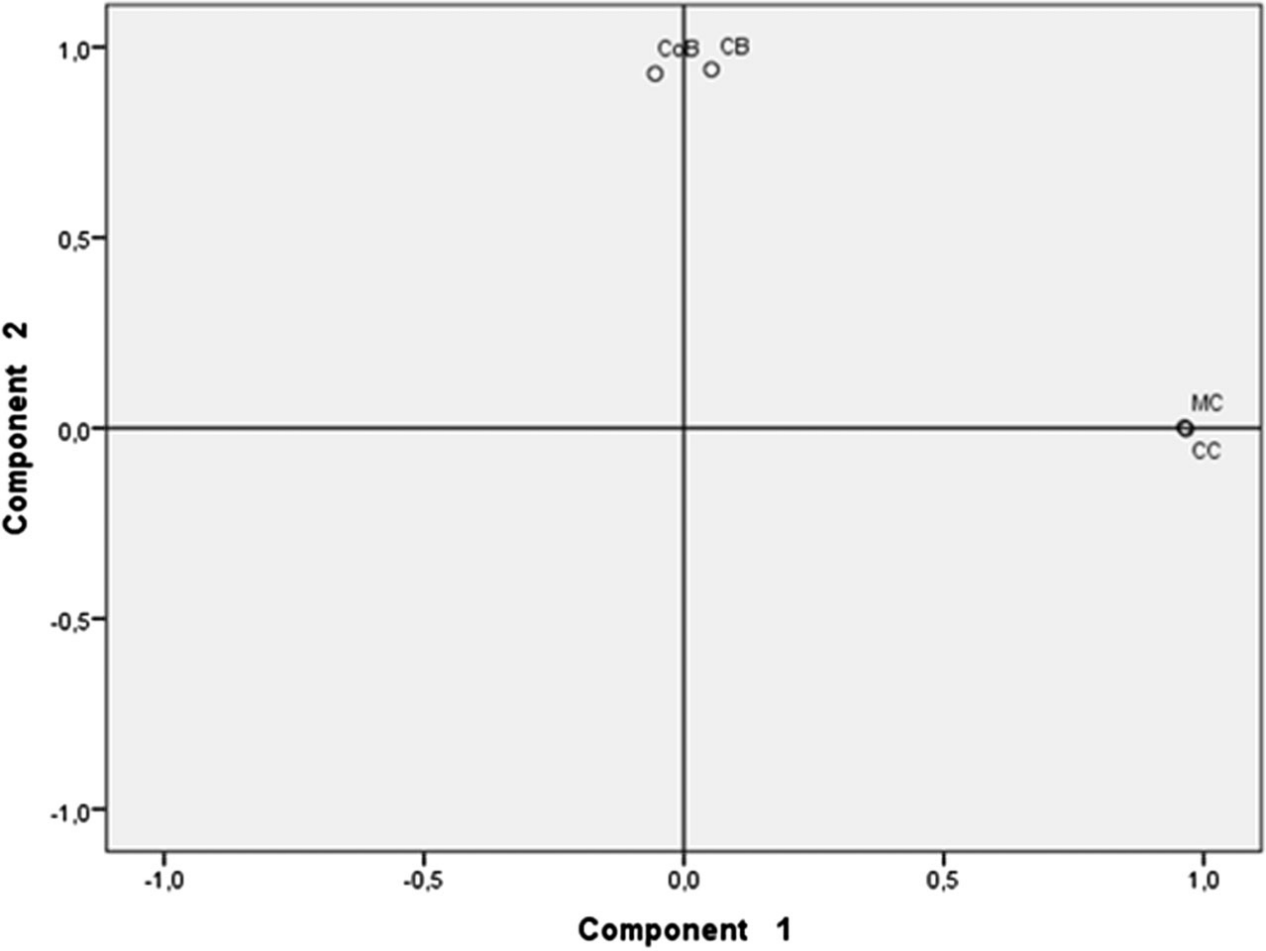
Principal component analysis of beers’ microbiota. Beers made with cassava (MC and CC) formed a cluster different from the cluster formed by beers made with either chonta (CB) or corn (CoB). Each pair of beverages that form a group share a similar bacterial species profiles and abundance.

The type of beer (fermented substrate) accounted for 90.4% of the bacterial species variability and cassava beers had more similar bacterial composition and abundance than the other types of beer; interestingly CB and CoB also showed similarity (Fig. 3).

## Discussion

Our study found higher bacterial diversity in beer that contained human saliva (Tables 2 and 3); therefore, saliva may not only speed up the fermentation process (by providing amylases as suggested by Henkel (2005)) but may also offer an additional bacterial inoculum which may favor this process. This finding may provide additional explanation for the adoption of such a peculiar process in the beer’s manufacture.

Our study also demonstrated the presence of oral streptococci such as *S. salivarius, S. mutans, S. parasanguinis* in cassava beers; these bacteria may thrive on carbohydrates present in the oral cavity after starchy meals (Moye, Zeng & Burne, 2014; Burne, 1998). Oral bacteria *S. salivarius* and *S. mutans* were cultured from cassava *chicha* (with saliva and without saliva) in large numbers and were shown to grow in mushed cassava under laboratory conditions. Oral bacteria in beer without human saliva may indicate contamination of fermenting containers (or utensils). Fermenting bacteria are known to produce biofilm in containers (Kebede et al., 2007) and both types of cassava beers were obtained from the same household, and most likely they use the same pots for both type of beers. It is possible that some strains of *S. salivarius* from these beers may be adapting to the fermentation process; *Streptococcus thermophilus*, a bacteria used as starter in yogurt (Burton et al., 2006) may have evolved from *S. salivarius* (Hols et al., 2005). Future studies should investigate the prevalence of *S. salivarius* in larger number of cassava *chichas* from other locations and find out whether the strains of *S. salivarius* isolated from beers are different from those isolated from human saliva.

A recent study failed to detect *S. mutans* and *S. salivarius* in *chicha* prepared with chewed cassava in Ecuador (Colehour et al., 2014). The disagreement between both studies may result from differences in samples in both studies; Colehour et al. (2014) collected beers that were fermenting for four days while we collected samples that were fermenting for three days. Beer microbiota changes overtime (Steinkraus, 2002) and in the case of *S. mutans* and *S. salivarius* we observed a sharp increase and decline in bacterial populations in 24 h (Figs. 1 and 2). Unlike Colehour et al. (2014), we also carried out bacterial cultures.

Reduction on streptococci populations may be due to the consumption of all the nutrients, accumulation of toxic metabolites, autolysis (Dufour & Lévesque, 2013). Also, these bacteria are known to form biofilm (Ajdić et al., 2002; Li et al., 2002) which may change bacterial location and reduction of planktonic cells. Additionally, unlike our study Colehour et al. (2014) found predominance of *L. reuteri* which is known to antagonize *S. salivarius* (Nikawa et al., 2004). Similar to previous studies (Colehour et al., 2014; Elizaquível et al., 2015; Puerari, Magalhães-Guedes & Schwan, 2015), *Lactobacillus* was a dominant genus of lactic bacteria in *chicha* found in both culture dependent and independent assessments.

Our study complements previous microbiological analyses carried out in *chicha* and shows for the first time the potential adaptation of *S. salivarius, S. mutants* (and possibly other streptococci from the human upper respiratory tract) to grow in cassava mush. The study not only shows how bacteria from human microbiota may adapt to artisanal fermentative processes but also shows that chewed *chicha* may potentially transmit human pathogens such as *S. mutans,* one of the causative agents of dental plaque and cavities (Loesche, 1986); *Streptococcus mutans* can be transmitted person-to-person, most likely through saliva (Baca et al., 2012). This is especially relevant because these types of beers are consumed as early as two or three days after preparation.

The main limitation of our study was the low number of samples analyzed of each beer. However this limitation does not invalidate the main findings of this study. Additionally, the culture medium (MRS) is not suitable to culture *Lactobacillus* from cereals (Minervini et al., 2012), therefore we may have underestimated the bacterial diversity in these beers.

## Additional Information and Declarations

### DNA Deposition

The following information was supplied regarding the deposition of DNA sequences: Genbank, accession numbers from KT722809–KT722833. SRA SRP070493; SRP070493, SRS1299611, SRX1612367, SRR3202831, SRS1299612, SRX1612366, SRR3202830, SRS1299613, SRX1612365, SRR3202829, SRS1310202, SRX1600290, SRR3187397, SRS1310203, SRX1600289, SRR3187396, SRS1310204, SRX1600288, SRR3187395, SRS1310207, SRX1612364, SRR3202828, SRS1310208, SRX1600292, SRR3202832.

### Data Deposition

The following information was supplied regarding data availability: The research in this article did not generate any raw data.
